# Perspectives of physicians and pharmacists on rational use of antibiotics in Turkey and among Turkish migrants in Germany, Sweden and the Netherlands: a qualitative study

**DOI:** 10.1186/s12875-022-01636-8

**Published:** 2022-02-15

**Authors:** Hilal Özcebe, Sarp Üner, Ozge Karadag, Achraf Daryani, Olga Gershuni, Katarzyna Czabanowska, Helmut Brand, Fabian Erdsiek, Tuğba Aksakal, Patrick Brzoska

**Affiliations:** 1 Department of Public, Faculty of Medicine, Hacettepe University, Ankara, Turkey; 2 Department of Public Health, Faculty of Medicine, Lokman Hekim University, Ankara, Turkey; 3 Columbia University, Earth Institute, Center for Sustainable Development, New York, NY USA; 4 Hacettepe University, Institute of Public Health, Ankara, Turkey; 5 Department of Public Health and Caring Sciences, Uppsala University, Uppsala, Sweden; 6 Department of International Health, Care and Public Health Research Institute – CAPHRI, Maastricht University, Maastricht, The Netherlands; 7 Department of International Health, Maastricht University, FHML, CAPHRI, Maastricht, The Netherlands; 8 Witten/Herdecke University, Faculty of Health, School of Medicine, Health Services Research, Witten, Germany; 9 Chemnitz University of Technology, Faculty of Behavioral and Social Sciences, Epidemiology Unit, Chemnitz, Germany

**Keywords:** Rational antibiotic use, Migrants, Germany, Sweden, Netherlands, Turkey

## Abstract

**Background:**

Antimicrobial resistance may result from inappropriate use of antibiotics in health care. Turkey is one of the countries with the highest antibiotic consumption in the world. Considering the role of transnational ties between Turkish migrants and their social contacts in Turkey, the attitudes and behaviors relating to rational antibiotic use in Turkey can also affect the use of antibiotics by Turkish migrants residing abroad. This study explores physicians’ and pharmacists’ experiences and perspectives on rational antibiotic use among Turkish adults in Turkey and among Turkish migrants in Germany, Sweden, and the Netherlands, three European countries with large populations of Turkish migrants.

**Methods:**

Following a qualitative study design using convenience and snowball sampling, in-depth interviews with 21 family physicians and 24 pharmacists were conducted in the aforementioned countries. We transcribed all interviews verbatim and performed content analysis separately in the countries, followed by translation, pooling and joint interpretation of the findings.

**Results:**

Physicians and pharmacists encountered irrational use of antibiotics among their patients in Turkey. Physicians interviewed in the three European countries explained that Turkish migrants differ from non-migrants with respect to their attitudes towards antibiotics, for example by more often expecting to be prescribed antibiotics. All physicians and pharmacists in the selected countries reported to inform their patients on how to use antibiotics upon prescription; however, Turkish migrants’ poor language proficiency was considered as a substantial communication barrier by the physicians and pharmacists interviewed in the European countries.

**Conclusions:**

The study illustrated some aspects of irrational antibiotic use among the population in Turkey and Turkish migrants in selected European countries. It emphasized the need for closer community participation, adequate information campaigns, as well as in-service training of health care providers in Turkey. The strategies and interventions on rational antibiotic use should also be supported and encouraged by health care providers, who need to reach out to people with various cultural backgrounds.

**Supplementary Information:**

The online version contains supplementary material available at 10.1186/s12875-022-01636-8.

## Background

High levels of antimicrobial resistance (AMR) have been reported by countries of all income levels. While the actual magnitude of antimicrobial resistance in humans is still unknown, it is estimated that AMR is responsible for at least 7 million annual deaths worldwide. This could increase to 10 million deaths if no further action is taken. Today, AMR is regarded as one of the important global public health problems [1].

Although the challenges with AMR have been known since antibiotics were first introduced into medical care, they are still the most used type of medicine worldwide [2]. AMR is linked to high consumption rates of antibiotics and other medicines and has gradually increased with population growth and rising levels of migration, travelling, urbanization and climate change [3–5]. Antibiotics have also been widely used in agriculture and livestock farming for the last decades [3, 4]. According to surveillance data from 65 countries, the World Health Organization (WHO) reports that overall consumption of antibiotics ranged from 4.4 to 64.4 Defined Daily Doses (DDD) per 1000 inhabitants per day, with strong variation between countries [6]. Klein et al. analyzed trends of antibiotic consumption between 2000 and 2015 and found that the antibiotic consumption rate increased by 39% (11.3–15.7 DDDs per 1000 inhabitants per day) in that period and estimated a further increase by 15% between 2015 and 2030 in case no policy changes are implemented [7].

AMR is the result of misuse and overuse of antibiotics by health institutions and communities [8]. According to the WHO, one cause of AMR is self-medication, which is particularly a problem in low and middle-income countries (LMICs), where antibiotics are often available from pharmacies without prescriptions [4]. Overprescription of antibiotics by healthcare professionals is another important factor that contributes to increases in AMR. Adjusting the prescribing behaviors of healthcare professionals concerning antibiotics is therefore considered an important intervention in this respect [9]. In addition to physicians, pharmacists are important stakeholders in consolidating efforts to support rational antibiotic use in communities [3, 10, 11].

Since research on new antibiotics has slowed down over the last years, the efficient use of available antibiotics is key to efforts aimed at reducing the development of AMR and improving infection control [12]. During the last year, the COVID-19 pandemic has led to an increase in antimicrobial consumption in hospitals and the prescription of broad-spectrum antibiotics can have an additional effect on AMR worldwide [13].

In the wake of the COVID-19 pandemic, dissemination of incomplete, inaccurate, or wrong information through mass and social media has led to confusion and inappropriate perceptions of health care services among people with limited health literacy, emphasizing the responsibility of health care providers to provide accurate information and treatment to their patients [14]. These developments have also led to anxiety, aggression, and medical mistrust in parts of the public, causing people to be afraid of being admitted to hospitals and preferring self-treatment at home [15, 16]. Another effect of the pandemic is the increased usage of hand sanitizers and soaps. The excessive use of hand sanitizers could increase AMR in the environment [17].

New policies and implementation strategies addressing AMR could be included into antibiotic stewardship programs [18]. Antimicrobial stewardship programs are becoming increasingly important due to possible effects of measures to prevent infections with SARS-CoV-2 on development of AMR. Therefore, understanding researchers’, health care providers’ and population perspectives on rational antibiotic use will be essential to improve strategies and measures of antimicrobial stewardship programs; that means optimal selection, dosage, and duration of antimicrobial treatments that result in the best clinical outcome for the treatment or the prevention of an infection, with minimal toxicity to the patient and minimal impact on subsequent resistance [19–21].

Studies show that antibiotics expenditure makes up an important share of healthcare spending in Turkey [22–24]. In 2015, four out of the six countries with the highest consumption rates in the world were LMICs, with Turkey being reported as the country with the highest antibiotic consumption [7]. The median consumption of antibiotics in the European region was reported by the WHO to be 17.9 DDD per 1000 inhabitants per day, whereas consumption in Turkey was more than twice as high (38.2 DDD) [6]. A meta-analysis estimated a pooled point prevalence of antimicrobial use of 45% and a median proportion of 36% inappropriate antimicrobial prescriptions in Turkey [25]. Low health literacy, high subjective demand for antibiotics, availability of antibiotics without prescription, sharing of antibiotics among family members or friends, and high self-treatment rates are the main causes of inappropriate use and overuse of antibiotics in the country [22, 23, 26].

In many developed countries throughout the world, migrants constitute large proportions of the respective populations. They are often vulnerable population groups which encounter several barriers resulting from poor language proficiency and from health care institutions not sufficiently catering to their cultural needs [27]. Immigrants from Turkey also make up large populations in several European countries, with more than 6 million people of Turkish descent living abroad, 5.5 million of which reside in Western European countries. Particularly large populations of Turkish migrants reside in Germany, the Netherlands and Sweden [28]. Over 3 million Turkish migrants are living in Germany [29], around 110,000 are living in Sweden [30] and approximately 400,000 are living in the Netherlands [31]. Turkish migrants in Germany have been reported to have a lower health literacy than other migrant populations in the country, particularly having difficulties in understanding doctors’ and pharmacists’ instructions on how to take prescribed medicines [29]. Yılmaz et al. also report language barriers and low health literacy as main barriers for Turkish-Dutch patients to understand medical information provided by health professionals and reference cultural beliefs as a barrier to informing cancer patients [32]. Another study found that language barriers and a low educational level among Turkish women were main factors of a higher diabetes prevalence compared to Swedish women [33]*.*

Although European countries have implemented comprehensive antimicrobial stewardship policies and regulations on rational antibiotic use, there are concerns that international migration contributes to AMR in Europe [34]. In addition, considering the role of transnational relations, the policies and prevalent attitudes towards the use of antibiotics in the country of origin may also affect the use of antibiotics by the migrant population residing abroad [34, 35].

Focusing on Turkish migrants as an example, the present qualitative study aimed to explore attitudes and behaviors of physicians and pharmacists regarding prescription or supply of antibiotics as well as their experiences and perspectives about rational antibiotic use among adults in Turkey and Turkish migrants in Germany, Sweden and the Netherlands. The study complements previous, including own, research which had been conducted on the perspective of migrants [36, 37].

## Methods

This descriptive study was part of a larger project to investigate irrational antibiotics use among Turkish adults and Turkish migrants in Europe from a patient and provider perspective. Therefore, all methodological decisions were made with respect to the overall project goal. For this study, we conducted partially structured qualitative interviews with Turkish people living in Turkey, Germany, Sweden and the Netherlands. Turkish immigrants make up particularly large shares of the population in these countries, while experiences with healthcare can vary greatly due to differences in demographic structure, structure of the healthcare system and history of immigration by Turkish immigrants in each country. Study sites in Germany, Sweden and the Netherlands were chosen for convenience and ease of access to potential participants [38, 39]. In total, we interviewed 21 physicians and 24 pharmacists (Table 1).

**Table 1:** The descriptive data of family physicians and pharmacists interviewed in the selected countries

Country	Family Physicians	Pharmacists
Turkey	11 physicians Age (min-max): 29–53 years Gender: 7 males, 4 females Professional experience (min-max): 5–29 years	9 pharmacists Age (min-max): 26–55 years Gender: 3 males and 6 females Professional experience (min-max): 3–30 years
Germany	4 physicians Age (min-max): 42–57 years Gender: 3 males and 1 female Professional experience: 10–28 years	5 pharmacists Age (min-max): 33–56 years Gender: 1 male and 4 females Professional experience: 10–31 years
The Netherlands	3 physicians Age (min-max): 44–50 years Gender: 2 males, 1 female Professional experience: 9–25 years	6 pharmacists Age (min-max): 26–53 years Gender: 1 male, 5 females Professional experience: 3–25 years
Sweden	3 physicians Age (min-max): 30–54 years Gender: 3 females Professional experience: 5–24 years	4 pharmacists age (min-max): 40–55 years Gender: 1 male and 3 females Professional experience: 10–25 years

### Data collection

Data were collected in 2016 and 2017 via semi-structured in-depth interviews with family physicians and pharmacists in the selected four countries to reflect the providers’ perspectives on rational use of antibiotics among citizens in Turkey and among Turkish migrants in Germany, Sweden and the Netherlands.

Two separate interview guides (see Supplement) were developed for family physicians and pharmacists based on previous research in the field [10, 19, 20, 24–26, 37]. The interview guides were developed in English, translated into the local languages of the participating countries by the researchers and pretested. The interview guide was used to explore the following topics: Physicians’ and pharmacists’ attitudes and behavioral patterns with respect to prescribing/supplying antibiotics, expectations and behaviors of patients concerning the prescription of antibiotics, physicians’ and pharmacists’ opinions on current problems and solutions regarding rational use of antibiotics among the Turkish population. We also collected information on sociodemographic characteristics of physicians and pharmacists such as age, gender, educational attainment, duration of professional experience and the duration of residence in current place of living.

Participants in Turkey, Germany and Sweden were recruited using a convenience sampling approach. In Turkey, in-depths interviews were conducted in an urban area of Ankara where the researchers were situated. Participants were working in different socioeconomic settlements determined by the Ankara Health Provincial Directorate. In Germany, participants were recruited in Bielefeld, North Rhine-Westphalia, where Turkish nationals account for ca. 6.4% of the population [40] and in Nuremberg, Bavaria, where Turkish immigrants account for 4.8% of the population [41]. In Sweden, recruitment took place in Uppsala, where people with a migrant background (born outside Sweden or both parents born outside Sweden) make up 28.4% of the population [42]. In the Netherlands, participants were recruited nationwide using a snowball sampling approach. This approach was chosen since recruitment efforts at the original study site Maastricht yielded no results.

In Germany, Sweden, and Turkey interviews were conducted in private homes, professional practices or office rooms in pharmacies, depending on the participants’ preferences. In the Netherlands, telephone interviews were conducted. The interviews took between 30 and 45 min. All participants were informed about the project goals and the interviewers’ professional background and gave informed consent to participate in the study. All interviews were audio-recorded. Aside from the audio recordings of the interviews, no further field notes were made during or after the interviews.

The interviews were conducted by researchers of the project (SÜ in Turkey, TA and FE in Germany, AD in Sweden and OG in the Netherlands). At the time of the study, SÜ was a professor for public health, while TA, FE, AD and OG were research assistants with several years of experience in the field. All researchers concerned with data collection are experienced in qualitative research techniques. There was no prior relationship between any of the interviewers and the participants.

None of the participants reached out to the research team afterwards, so no feedback on the findings was provided by respondents.

### Data analyses

For our analysis we used directed qualitative content analysis. Predetermined main themes based on the interview guide were used to structure the content analysis. Main themes for family physicians were “patients’ rational antibiotic use behaviors”, “prescription of antibiotics” and “informing patients on rational antibiotic use”, whereas the themes for pharmacists were “prescription patterns and request of antibiotics without prescription”, “delivery of information on rational antibiotic use” and “perceived problems and recommendations with respect to rational antibiotic use”. These guided the coding process, supplemented with inductively generated codes for statements that did not fit the initial codes. Qualitative data were transcribed verbatim in the respective languages they were collected in and initial coding was performed by the data collectors separately in each country, in order to allow an analysis based on the native language of the interview. Codes were translated, pooled and jointly interpreted by the entire international research team following established guidelines [43].

## Results

In total, 21 family physicians (11 in Turkey, 4 in Germany, 3 in Sweden, and 3 in the Netherlands) were interviewed. Physicians’ professional experience ranged between 5 and 29 years in Turkey, and between 5 and 28 years in the European countries. The total numbers of pharmacists interviewed were 24 (9 in Turkey, 5 in Germany, 6 in the Netherlands and 4 in Sweden) and their professional experience ranged between 3 and 30 years in Turkey and between 3 and 31 years in the European countries (see Table 1).

The findings can be clustered into two main subgroups: 1) Physicians’ experiences with respect to rational antibiotic use of their patients and their experience concerning prescribing antibiotics and 2) pharmacists’ experiences regarding rational antibiotic use and their communication with Turkish patients***.***

### Physicians’ experience on rational antibiotic use of their patients and their practice of prescribing antibiotics

#### Inappropriate antibiotic use among patients 

Participating physicians in Turkey indicated that attitudes and behaviors of Turkish patients with respect to antibiotic use were generally unfavorable, being characterized by patterns such as using antibiotics without prescription, stopping to take antibiotics after disappearance of symptoms, not attending scheduled appointments with physicians when symptoms were no longer experienced, and taking advice on antibiotic use from family members and/or friends.

*“The person decides on his/her own that he/she is sick. He/she decides that this disease will be cured with antibiotics. He/she obtains that antibiotic and discontinues using it when his/her complaints regress in 2 or 3 days. There are people, who use a single tablet!”*

*(Family physician, Turkey)*

Main problems perceived by German physicians concerning irrational antibiotics use among Turkish migrants were related to dosages and the adherence to treatment. According to the physicians in Germany, Turkish migrants frequently stopped taking antibiotics once they were free of symptoms. In addition, left-over medicines were sometimes shared with friends and relatives. In the Netherlands, participants, similarly, stated that Turkish migrants discontinued treatment after feeling better.

*“They say: ‘I was feeling better and so I stopped taking them [the antibiotics]’.”*

(Physician, Germany)

*“Sometimes they say: ‘well, my neighbor had some antibiotics left and he gave them to me, so I took them.’”*

(Physician, Germany)

*“If they feel better, they might discontinue treatment.”*

(Family physician, the Netherlands)

#### Patients’ requests concerning prescription of antibiotic by physicians

The physicians in Turkey emphasized that, although patients’ requests for prescriptions for antibiotics have declined in recent years, such requests are still common in daily practice. Family physicians in Turkey stated there was still a need to raise awareness among the public with respect to rational antibiotic use.

*“I think that the solution to this problem would be to provide non-formal education to the public, .... It would be very helpful that public education on what an antibiotic is and when it is required should be given through physicians or other health professionals who are members of the staff of the organization or a medical group, or through public service announcements.”*

*(Family physician, Turkey).*

Family physicians in Germany, Sweden and the Netherlands stated that Turkish migrants usually shared their opinion about possible treatment options, similar to non-migrant patients. German physicians emphasized that the majority of patients had largely good knowledge about antibiotics and did not have many questions when antibiotics were prescribed. Interviewees in Germany also emphasized that especially younger age groups (second-generation migrants) had good knowledge about side effects and multi-drug resistance. Physicians in both Germany and the Netherlands, however, also considered Turkish migrants to request antibiotic prescriptions more often than the respective majority populations.

*“If they will come with some minor inflammatory reaction or even a flu, they often expect to start using antibiotics. This pattern is certainly different compared to the general population”.*

*(Family physician, the Netherlands)*

#### Prescribing behaviors of physicians

Family physicians in Turkey reported that they did not prescribe antibiotics based on patients’ requests. They also stated that they decided to prescribe antibiotics as a therapeutic regimen based on their clinical experience (anamnesis, inspection and physical examination) rather than requesting blood tests or cultures for confirming a microbial infection.

*“I am telling you in good language that I cannot prescribe antibiotics to any patient without an examination. But despite this, when they insisted, we started very urgently, especially on the weekend. I speak at length to those who say that they cannot be cured with any medicine other than antibiotics, sincerely and without prejudice, without doing anything. I explain that the use of antibiotics should not be abused so much, that the patient should not decide on the treatment.*

*(Family physician, Turkey).*

*“We lack of performing laboratory tests in the primary care setting. I can obtain the results of the tests I asked for today the next day around 1.30 PM. Therefore, I often make a decision based on physical examination findings and medical history of the patient”.*

*(Family physician, Turkey).*

Physicians in Germany, the Netherlands and Sweden were generally very cautious about when to prescribe antibiotics. Physicians in Sweden stated that they were following guidelines, used diagnostic tools correctly and stated to rarely feel unsure about their decision to prescribe antibiotics. They added that they were usually in agreement with the patient about possible treatments and procedures. Dutch physicians often used C-reactive protein (CRP) blood testing in order to determine whether an antibiotic treatment is indicated [18]. This procedure was reported to be often used for migrant patients in order to show and in some cases to convince those patients that antibiotics are not needed or not effective in their particular case. The interviewed physicians in Germany stated that their prescribing behaviors did not differ between migrant and non-migrant patients.

*“Your CRP value is X and it will take some time for you to get better, however according to this value taking antibiotics makes no sense at all”; “Our patients are already used to it”; “It will be generally accepted if the doctor explains, our patients will not insist”.*

*(Physician, the Netherlands)*

Apart from this, some physicians stated that Turkish migrants were more likely to ask for antibiotics and would have a less critical/skeptical attitude towards using antibiotics than the general German population. Dutch physicians also emphasized that patients with a Turkish background would ask directly if they “s*houldn’t use antibiotics to get better”.*

“*We have quite some Turkish patients in our practice. If they will come with some minor inflammatory reaction or even a flu and they often expect to start with antibiotics. This pattern is certainly different compared to general population”*.

*(Physician, the Netherlands)*

Patients’ expectations are considered by physicians interviewed in Germany to be largely based on positive prior experience with specific medicines, including both personal use and experience of close friends, relatives or other trusted persons. Also, in some cases, Turkish patients – mainly older patients – in the Netherlands would bring (empty) medication boxes from Turkey back to their physicians in the Netherlands.

*“I was not feeling well, and the doctor prescribed me this. It really helped me!”; “It can be expected from me that I will prescribe the same drug”.*

*(Physician, the Netherlands)*

*“With migrant patients, it is like: a cold always means germs, bacteria, there is no differentiation between viruses and bacteria, and they don’t know, what antibiotics are for. They say:,This always helps.’ Like some kind of panacea.*“.

*(Physician, Germany)*

#### Informing patients on rational antibiotic use

Family physicians in Turkey reported that they provided verbal information to their patients on how to use the antibiotics prescribed. However, they believed that patients with low and middle socioeconomic status either did not understand the information or did not want to continue using antibiotics after feeling better.

*“I also think that 20%, maybe 30% of the patients do not fully complete the recommended course of the drug treatment. I mean, this is so, because in the next visit, the patient tells me that he/she has this/that drug at home already. You know what I’m saying? This suggests that he/she did not use the drug when I previously prescribed it, if there are 10 tablets, he/she used only 6 of them and still has 4.”*

*(Physician, working at low socioeconomic settlements, Turkey)*

“*… … when I prescribe antibiotics, I say: ‘Finish it all. I’m trying to tell you to finish the whole box or use it for this many days or something.’ But for some reason, the next time they come with another illness, they tell me they have already taken the antibiotics that I prescribed the last time …*”.

*(Physician, working at medium socioeconomic settlements, Turkey)*

Physicians in the Netherlands and Sweden reported that the relevant case-specific information was provided verbally to patients during the consultation. Physicians in Germany stated that the information provided was mostly related to potential side effects and instructions for administration/application of the medicine. According to one physician, patients of Turkish origin mostly did not adopt an active patient role.

*“They don’t actively ask. Either I tell them immediately how they should take it or I write it down.”*

*(Physician, Germany)*

Physicians in all three European countries agreed that providing written information was very important for patients. Due to language barriers, the importance of multi-language brochures was emphasized. However, brochures on rational antibiotic use were not always available. Dutch and Swedish physicians considered Turkish patients with sufficient language proficiency to understand the written materials. Physicians in the Netherlands indicated websites could also be accepted as health information resources for migrants.

### Pharmacists’ experiences regarding rational antibiotic use and their communication with Turkish patients

#### Patients’ requests

Pharmacists in Turkey reported that, compared to previous years, family physicians are now better trained, and legal restrictions on prescriptions were effective in increasing awareness about rational use of antibiotics. On the other hand, there is also a belief that family physicians still sometimes prescribe broad-spectrum antibiotics without any clinical need.

*“Antibiotic prescribing decreased. To most of the patients that asked, we already said: ‘We can’t prescribe antibiotics. You know, a control mechanism was introduced for family physicians.’”*

*(Pharmacist, Turkey)*

*“I believe that the physicians should inform patients on antibiotic use. Unfortunately, family physicians in particular, really like prescribing antibiotics. Without doing anything, without examining the patient, without even mentioning any laboratory tests, they like prescribing antibiotics”.*

*(Pharmacist, Turkey)*

The pharmacists in Turkey also reported that they never sold antibiotics without prescription because they knew it was illegal in the country. They further explained that although patients’ requests for antibiotics without prescription have declined in recent years, they still continue to receive such requests frequently, especially in case of respiratory tract infections. They believed that there was a need to raise awareness among both family physicians and the general public to increase rational antibiotic use.

*“Despite the recently enacted prohibition against selling antibiotics without prescription in Turkey, patients are unbelievably insistent. They absolutely do not want to visit a physician. Even in the smallest problems, such as influenza, they ask for an antibiotic.”*

*(Pharmacist, Turkey)*

There were no reports about antibiotics being provided without prescriptions in any EU country included in the study. Pharmacists in Germany, Sweden and the Netherlands agreed that Turkish migrants had similar behavioral patterns as the respective majority populations concerning rational use of antibiotics. Only a small portion of migrant customers would knowingly ask for antibiotics without a prescription in Germany and the Netherlands according to the interviewees. The pharmacists stated to not sell antibiotics without a prescription, since this is illegal and could lead to serious consequences for the health and safety of the patients. Most of the pharmacists interviewed reported having doubts about patients actually knowing about the applicability of antibiotics in their particular case and whether a specific drug is an antibiotic.

*“It is very, very rarely that –that they ask directly for antibiotics. By far, in most of the cases they just hand us a prescription from a doctor. And usually they don’t demand or expect a specific branded product.”*

*(Pharmacist, Germany)*

#### Delivery of information on rational antibiotic use at the pharmacies

Pharmacists in Turkey mostly reported that they deliver information to their patients about rational antibiotic use and emphasized the general need for doing so.

*“I explain even if it is written in the prescription, that they have to be really careful about taking the medication, particularly about the time, and that they should not discontinue the medication earlier than the recommended duration.”*

*(Pharmacist, Turkey)*

Providing information on rational antibiotic use was considered the main subject of communication between pharmacists and Turkish migrants in Germany, Sweden and the Netherlands when they approached pharmacies to fill a prescription for antibiotics. Communication was focused on the treatment duration, the prescribed dosage and side effects. The pharmacists pointed out that in order to overcome language barriers, they usually ask accompanying relatives of patients, other customers, or other staff for assistance if needed. Despite existing language barriers, pharmacists reported that Turkish language information materials on rational antibiotic use were usually not readily available.

*“After all, there are enough people who speak both languages and then we just wait until someone comes in and then they can translate.”*

*(Pharmacist, Germany)*

*“We will provide extensive information regarding different aspects of antibiotics consumption (side-effects and what they may encounter, etc.) and this will also be recorded in our system … If the antibiotics have to be delivered at home, we will call those patients and will provide the same information by phone.”*

*(Pharmacist, the Netherlands)*

The findings further showed that younger migrants in the European countries, especially the second generation of Turkish migrants, were more cautious about the use of antibiotics and would ask more questions regarding the dosage and the duration of the prescribed drugs.

*“The second generation is definitely healthier and more aware of rational use”.*

*(Pharmacist, the Netherlands)*

#### Transnational practices of antibiotic use among Turkish migrants

Pharmacists in Turkey reported that it was not uncommon for Turkish migrants’ who visit Turkey to take antibiotics with them when returning to their respective countries of residence.

*“It has been three summers. A customer arrived, and told me that he wanted to purchase, like, 10 packs of antibiotics. I asked why, and he said: “I am living in the UK. They do not sell antibiotics there. It is neither prescribed, nor sold. We suffer from fever for two to four days, and even then, they do not sell antibiotics. I am thinking of buying the antibiotics from here …*. ”.

*(Pharmacist, Turkey)*

Furthermore, some physicians learned that their patients used antibiotics in Turkey and did not inform their physicians in Germany and the Netherlands. Also, physicians reported that some Turkish migrants also used antibiotics which they brought from Turkey.

*“In Turkey they can get antibiotics easily and will not always inform their GP here in the Netherlands”.*

*(Family physician, the Netherlands)*

*“One of my patients brought some combination of wide spectrum antibiotics back to the Netherland for her urinary tract infection.. …” .*

*(Family physician, the Netherlands)*

## Discussion

This study showed that, from the perspectives of family physicians and pharmacists, there were different expectations and behaviors on rational antibiotic use among Turkish citizens in Turkey when compared to Turkish migrants in Europe. Family physicians in Turkey frequently encountered irrational use of antibiotics among the local population. Likewise, physicians interviewed in the three European countries explained that Turkish migrants have irrational behaviors with respect to antibiotic use and expect antibiotic prescriptions more often than the respective majority populations.

The World Health Organization (WHO) highlights the critical importance of rational antibiotic use and calls for adequate education of healthcare staff and the public with respect to the appropriate use of antibiotics as part of a concerted action against antimicrobial resistance [44]. The first program in Turkey was introduced in 2003 to decrease antimicrobial use at hospitals, requiring preauthorization from an infectious disease specialist ahead of treatment with several broad-spectrum antibiotics. The second program was implemented in 2014 to reduce antimicrobial prescription in primary health care units, especially for acute respiratory infections. Health care providers working at primary health care units were trained on rational antibiotic prescription and a public awareness campaign was conducted through mass media in the country. In addition, the rapid strep test was implemented at primary health care facilities. The rapid test is used to diagnose upper respiratory tract infections with group A streptococcus. The Turkish government also introduced new regulations in reimbursement schemes to address the growing problem of irrational use of antibiotics. In this regulation system, control was strengthened by establishing a prescription information system which aims to follow up unnecessary antibiotic prescriptions. Also, a reporting system was introduced which describes the indicators of antibiotics prescriptions by the specialty of physicians. Furthermore, over-the-counter sales of antibiotics without prescriptions were banned and campaigns on increasing awareness of rational antibiotic use in the community are conducted [45]. With these interventions, physicians’ adherence to these regulations of antibiotic prescriptions is supposed to improve and the increased supervision by the Ministry of Health aims to make the purchase of antibiotics from pharmacies without a prescription impossible [26, 46]. The proportion of antibiotics among all prescriptions issued by family physicians decreased to 25% in 2017 [45]. Despite data showing a declining trend in overall consumption of antibiotics after introduction of new policies and regulations, antibiotics consumption is still higher than actual need and the irrational use of antibiotics remains a significant public health concern among the population in Turkey [46]. There is evidence that citizens still do obtain antibiotics without prescription and keep them for future use [47–49]. Our study also showed that physicians and pharmacists emphasized the relevance of the new regulation on the prescription of antibiotics. However, they believed that misuse, overuse and inappropriate antibiotic use was still a problem in the community as patients frequently attempted to get prescriptions for antibiotics from physicians or to buy them without prescription from pharmacists. In contrast, physicians declared that they prescribe antibiotics based on the clinical history of the patients and physical examination. In our study, none of the pharmacists reported selling antibiotics without prescription, but they also mentioned that it was possible to buy antibiotics without prescription in the country. This suggests that despite regulations on rational antibiotic use in Turkey, attitudes and behaviors on rational antibiotic use have not yet changed among all health care providers. This is in line with previous studies in the field [48, 49].

Responding to AMR is considered to be a priority issue by the European Commission, and an action plan was prepared to address the threats arising from antimicrobial resistance [35]. An effective antimicrobial policy was implemented through interventions such as antimicrobial stewardships, public health campaigns, improved diagnostics and revised guidelines in many countries [11]. In addition, surveillance systems on AMR and antibiotic consumption were set in place. The total antibiotic consumption rate in European countries decreased by 20–30%, and inappropriate prescription was reduced by more than 50% in the Netherlands between 2015 to 2019 [9]. In the present study, physicians in Germany, Sweden and the Netherlands indicated that they mostly used laboratory tests before prescribing antibiotics, and referenced national guidelines from antibiotic stewardship programs in Europe. National rules were also referenced by pharmacists in these countries. Health care providers reported that most Turkish migrants showed similar behaviors as the majority population; but also mentioned that in some cases Turkish migrants asked physicians to prescribe antibiotics and pharmacists to sell antibiotics without prescription. Besides sometimes misplaced perceptions about the effectiveness of antibiotics, social factors and culturally informed behaviors are known to be relevant reasons for irrational antibiotic use in the communities [8, 10, 50].

Physicians in Europe stated that Turkish migrants in some cases brought antibiotics from Turkey; which was also corroborated by pharmacists interviewed in Turkey. This shows that Turkish migrants could use antibiotics without prescription in the host countries which is a risk for the development of AMR in European countries. Nellums et al. reported high AMR rates among refugees and asylum seekers; but also pointed out that further information about other migrant groups is needed [34]. Our study showed that health care providers were mostly aware of the influence of their cultural infrastructure on antibiotic use among Turkish migrants.

Communication between health care providers and migrants may be affected by demographic and socioeconomic factors, the cultural background and the level of language proficiency [51, 52]. According to a study by Würth et al. conducted in Switzerland, Turkish migrants reported difficulties in following doctors’ suggestions, especially concerning medication, self-monitoring and adhering to follow-up appointments [53]. This is confirmed by other studies from Germany and the Netherlands highlighting problems with treatment and medication adherence due to social, cultural and healthcare access factors [54, 55]. Bermejo et al. showed that Turkish migrants in Germany frequently encounter language-related barriers in healthcare services, and report feeling misunderstood regarding their cultural needs by the healthcare professionals [56]. In our study, language or cultural factors were also mentioned as significant barriers faced by health care providers in Germany, Sweden, and the Netherlands. Physicians and pharmacists also emphasized that particularly first generation Turkish migrants were hesitant in asking questions. This could be because of insufficient language skills. However, although interviewees considered many first-generation Turkish migrants to have poor language proficiency, this was mostly not regarded as a problem due to the reliance on family members and other lay interpreters with proficiency in Turkish and the respective language of the host country. Many Turkish migrants have lived in the respective host countries for many decades, together with their families and often embedded in sizable migrant communities. Therefore, lay ad-hoc interpreters are often easily accessible for health care professionals in case of communication problems. Reliance on support from lay interpreters is associated with many challenges though, such as potentially incorrect translations and limited confidentiality [57]. In contrast to first generation migrants, second generation migrants were reported to experience less barriers and to fare better in terms of rational use of antibiotics. This highlights the heterogeneity of this population group which also needs to be taken into account by measures of health education.

### Implications

It is estimated that the relevance of AMR as a public health problem will further increase in the future as a result of developments from the current COVID-19 pandemic [13, 17]. Infodemics on social media experienced during COVID-19 have affected the mental health and health care seeking behaviors of health care users [15, 16], suggesting a high importance of health care professionals as providers of scientifically sound and accurate information and treatment for their patients [14, 37]. In our study, we found that physicians and pharmacists in Turkey believed that there was a need for community information campaigns as well as in-service training for health care providers on rational antibiotic use. Physicians in European countries emphasized that providing written documents was an important aspect of informing Turkish migrants; which was also addressed by the pharmacists interviewed. Written documents on rational antibiotic use should include information on further reliable sources and promote healthy behaviors.

### Strengths and limitations of the study

Main strengths of this study are its comparable recruitment strategy used in four different countries and the joint analysis of the data by an international research team. In addition, integrating perspectives from physicians and pharmacists has allowed us to consider interprofessional communication problems and provide a more comprehensive analysis of the topic. Our transnational approach has further added to the relevance of our findings, providing the foundations for future quantitative studies aiming to address differences in antibiotics use between Turkish migrants and the population in Turkey, as well as between Turkish migrants in different host countries.

The main limitation of this qualitative research was that interviews were conducted only in selected regions of the respective countries. Information on recently migrated individuals was lacking, which could have been important for identifying adaptation processes and for examining the time dependency of existing attitudes and patterns of antibiotic use. The sample size of the current study was comparatively small. However, the collection of further data did not provide any new findings. Given the saturated data, we do not consider the sample size to be a limitation.

## Conclusions

The qualitative present study identified different attitudes towards practices of rational antibiotic use among citizens in Turkey when compared to Turkish migrants in the selected European countries from the perspectives of family physicians and pharmacists. The results of this study gave us some important detailed evidence that could not have been learned by quantitative research techniques. It particularly showed the need to increase awareness of the importance of rational antibiotic use in Turkey. In European countries, the strict implementation of rules on rational antibiotic prescription contributed to a more cautionary use of antibiotics and respective health seeking behaviors of Turkish migrants. Findings showed that language barriers still exist and communication between health care providers and first-generation Turkish migrants remains a challenge. Therefore, appropriate strategies, including availability of professional interpreters as well as informational materials in Turkish, will likely significantly improve health information and health care access for this population group in the respective host countries.

## Supplementary Information

**Additional file 1.** Supplement for “Perspectives of physicians and pharmacists on rational use of antibiotics in Turkey and among Turkish migrants in Germany, Sweden and the Netherlands: A qualitative study “(Ozcebe et al.). Guides used for qualitative interviews.

## Data Availability

All transcript used for the analysis are available from the corresponding author upon request.

## Abbreviations

AMR: Antimicrobial resistance
DDD: Defined Daily Doses
LMICs: Low middle income countries
WHO: World Health Organization
