# Supplementary material for: Perspectives of physicians and pharmacists on rational use of antibiotics in Turkey and among Turkish migrants in Germany, Sweden and the Netherlands: a qualitative study
Source: BMC Prim Care. 2022 Feb 15;23:29. doi: 10.1186/s12875-022-01636-8 (PMC8848838; doi:10.1186/s12875-022-01636-8)
Supplement: Supplementary file 1 — Additional file 1. Supplement for “Perspectives of physicians and pharmacists on rational use of antibiotics in Turkey and among Turkish migrants in Germany, Sweden and the Netherlands: A qualitative study “(Ozcebe et al.). Guides used for qualitative interviews. [file 12875_2022_1636_MOESM1_ESM.pdf]

Supplement for *“Perspectives of physicians and pharmacists on rational use of antibiotics in Turkey and among Turkish migrants in Germany, Sweden and the Netherlands: A qualitative study“* (Ozcebe et al.)

Guides used for qualitative interviews

Interviews with physicians

Physicians in Sweden, Germany and the Netherlands:

- How do Turkish migrants ask for antibiotics? Do they ask for antibiotics without prescription? Do they know the brand name? Do they ask about dose and duration of antibiotics?
- If you refuse to prescribe the antibiotics, how do they respond? Do they insist on getting the antibiotics?
- How do you provide information on how to use antibiotics correctly?
- What can you say about Turkish migrants’ adherence to prescribed antibiotics regimens?
- Are migrants’ attitudes towards antibiotics different from the attitudes of non-migrant patients or migrant patients of other descent?
- Have you ever had the feeling that the medicines you prescribe to your Turkish migrant patients interfere with regimens they obtain while travelling to Turkey?

Physicians in Turkey:

- How do Turkish people ask for antibiotics? Do they ask for antibiotics without prescription? Do they know the brand name? Do they ask about dose and duration of antibiotics?
- If you refuse to prescribe the antibiotics, how do they respond? Do they insist on getting the antibiotics?
- How do you provide information on how to use antibiotics correctly?
- What do you think on rational antibiotic usage in Turkey? What kind of problems have you encountered? Does the Social Security Institution provide any regulation?

Interviews with Pharmacists

Pharmacist in Sweden, Germany and the Netherlands:

- How do Turkish migrants ask for antibiotics? Do they ask for antibiotics without prescription? Do they know the brand name? Do they ask about dose and duration of antibiotics?
- In case they ask for antibiotics without prescription: If you refuse to sell the antibiotics, how do they respond? Do they insist on getting the antibiotics?
- How do you provide information on how to use antibiotics correctly?

Pharmacist in Turkey:

- How do Turkish people ask for antibiotics? Do they ask for antibiotics without prescription? Do they know the brand name? Do they ask about dose and duration of antibiotics?
- If you refuse to sell the antibiotics, how do they respond? Do they insist on getting the antibiotics?
- How do you provide information on how to use antibiotics correctly?
- What do you think on rational antibiotic usage in Turkey? What kind of problems are health professionals confronted with? Does the Social Security Institution provide any regulation?
